# Pneumothorax with Ortner syndrome: an unusual presentation of aortic dissection

**DOI:** 10.1002/rcr2.718

**Published:** 2021-02-15

**Authors:** Shan Min Lo, Hema Yamini Ramarmuty, Kunji Kannan

**Affiliations:** ^1^ Department of Respiratory Hospital Queen Elizabeth Kota Kinabalu Malaysia

**Keywords:** Aortic dissection, Ortner syndrome, pneumothorax, recurrent laryngeal nerve palsy

## Abstract

Ortner syndrome or cardiovocal syndrome is hoarseness of voice due to left recurrent laryngeal nerve palsy as a result of cardiovascular abnormality. It is not known that pneumothorax has any association with Ortner syndrome. A 56‐year‐old gentleman, with previous history of 20 pack‐year smoking and 1‐year history of hypertension, presented to us with cough for two weeks with intermittent haemoptysis, as well as hoarseness of voice for the past one year. Direct laryngoscopy confirmed that he had left vocal cord palsy. Clinical and radiological investigations suggested that he had left pneumothorax. Left chest tube thoracostomy was performed and computed tomography of chest revealed aortic isthmus aneurysm with dissection extending to distal left common iliac artery and residual left hydropneumothorax. The patient was then referred to the vascular team and cardiothoracic team for further management.

## Graphical abstract

This study describes pneumothorax with Ortner syndrome, which is an unusual presentation of aortic dissection.

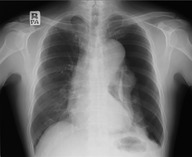

## Introduction

There are few published articles showing that patients with aortic dissection may present with Ortner syndrome. However, to date, there is no published article regarding Ortner syndrome association with aortic aneurysm and pneumothorax. Clinical presentation of aortic aneurysm and pneumothorax can be both variable, ranging from non‐specific symptoms to cardiorespiratory collapse. Both diseases are potentially life‐threatening and require immediate medical attention. This case reports a man who presented with a first episode of spontaneous left pneumothorax with incidental finding of aortic dissection on computed tomography (CT) of the chest. We were able to both identify the life‐threatening medical condition and manage the patient accordingly.

## Case Report

A 56‐year‐old gentleman was referred to our centre from a nearby district hospital with cough for two weeks associated with intermittent haemoptysis. He denied chest pain or dyspnoea. He did not report any constitutional symptoms. He also complained of hoarseness of voice for the past one year. He has a background history of hypertension diagnosed a year back. He is an ex‐smoker with 20 pack‐year smoking history. His family history was unremarkable. Chest radiograph at the district hospital showed a left‐sided pneumothorax; therefore, he was subsequently transferred to our centre.

**Figure 1:**
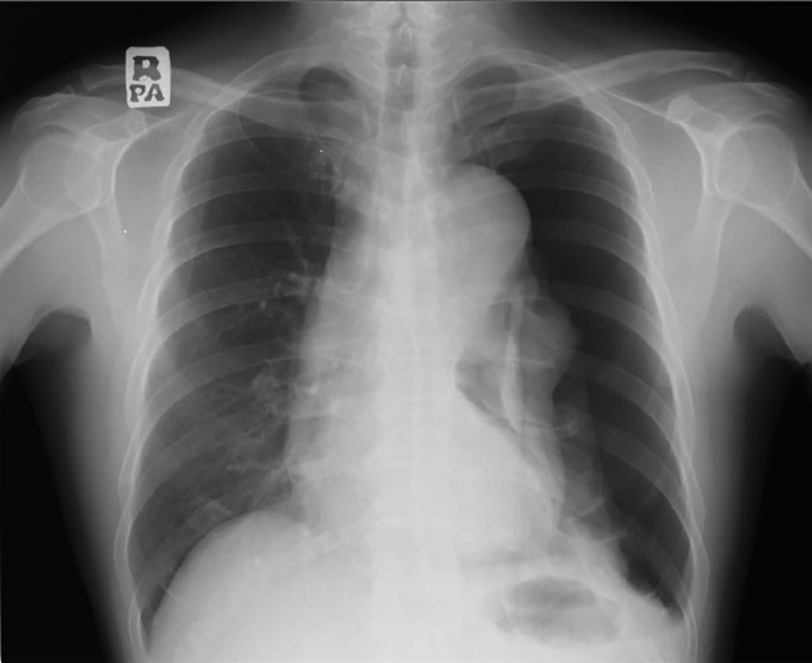
Chest radiograph showed left pneumothorax without trachea deviation.

On clinical presentation, his blood pressure (BP) was 169/106 mmHg, heart rate 91 beats per min, and his peripheral capillary oxygen saturation (SpO_2_) was 96% under room air. He was not in respiratory distress. Chest auscultation revealed reduced air entry over the left lung. His trachea was not deviated. He had no signs to suggest Marfan syndrome. Chest radiograph revealed a left pneumothorax (Fig. 1). Left chest tube thoracostomy was performed. Direct laryngoscopy was performed by otorhinolaryngology team which confirmed that the patient has left vocal cord palsy. Another important point to note was that the patient's blood pressure ranged 140–150/90–100 mmHg despite being on three antihypertensive agents.

**Figure 2:**
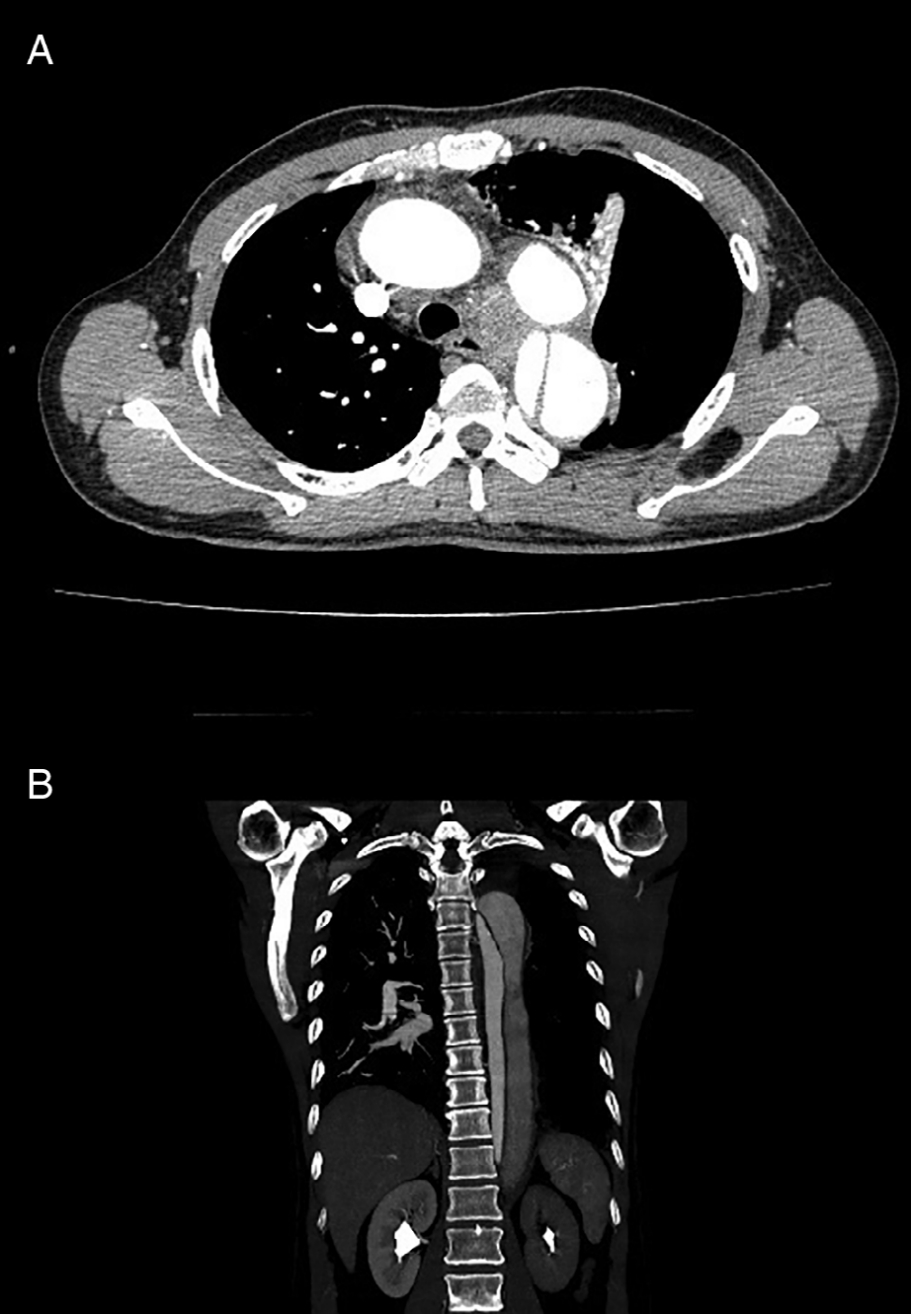
(A) Aortic aneurysm below the arch of aorta with haematoma within the mediastinum. (B) Aortic isthmus aneurysm with dissection extending to the distal left common iliac artery with no evidence of aortic rupture.

CT of the chest was performed in view of unexplained hoarseness of voice with spontaneous pneumothorax. Surprisingly, it showed residual left hydropneumothorax as well as an aortic dissection, distal to the origin of the left subclavian artery complicated with haematoma within the mediastinum and an aneurysm inferior to the arch of aorta (Fig. 2). A full CT angiogram (CTA) was subsequently performed and showed aortic isthmus aneurysm with dissection extending to distal left common iliac artery with no evidence of aortic rupture (Fig. 2). The patient was then referred to the vascular team and cardiothoracic team for further surgical management.

## Discussion

There are few published articles regarding patients with aortic dissection presenting with Ortner syndrome [1, 2]. Our patient also had hoarseness of voice for the past one year. From the CT thorax and CTA, it is suggestive that this patient had left recurrent laryngeal nerve palsy due to compression from the aortic aneurysm. This suggests that he may have had an asymptomatic chronic aortic aneurysm for the past one year or longer.

With the new‐onset of left‐sided pneumothorax, this patient might be having two different pathologies occurring concurrently or an aortic dissection as a result of his pneumothorax.

On further literature search, we have found two published case reports regarding type A aortic dissections occurring following tension pneumothorax [3, 4] and a case report of aortic dissection associated with pneumothorax [5]. One of the case reports [4] concluded that their patient developed aortic dissection following pneumothorax, because progressive pneumothorax increased intrathoracic pressures to such extent as to cause aortic dissection with an aortic aneurysm. This could be the case in our patient.

In conclusion, although it is a rare entity, aortic dissection may be associated with pneumothorax and should be considered especially if the patient's condition does not improve even after decompression of the pneumothorax. Clinicians also need to have high degree of clinical suspicious when symptoms are not explainable with the apparent clinical diagnosis.

### Disclosure Statement

Appropriate written informed consent was obtained for publication of this case report and accompanying images.

### Author Contribution Statement

Lo Shan Min designed the work and interpreted the data. Hema Yamini Ramarmuty revised the manuscript critically for important content. Kunji Kannan is responsible for the final approval of the version to be published.
